# Level, Trend and Correlates of Mistimed and Unwanted Pregnancies among Currently Pregnant Ever Married Women in India

**DOI:** 10.1371/journal.pone.0144400

**Published:** 2015-12-02

**Authors:** Mili Dutta, Chander Shekhar, Lokender Prashad

**Affiliations:** 1 Department of Fertility Studies, International Institute for Population Sciences, Mumbai, India; 2 School of Social Sciences, Tata Institute of Social Sciences, Mumbai, India; University of Rochester, UNITED STATES

## Abstract

Unintended pregnancy accounts for more than 40% of the total pregnancies worldwide. An Unintended pregnancy can have serious implications on women and their families. With more than one-fourth of the children in India born out of unintended pregnancies such pregnancies are considered to be one of the major public health concerns today. The present study is aimed at determining major predictors of unintended pregnancy among currently pregnant ever-married women in India. The present study has used National Family Health Survey (NFHS) data, conducted by the International Institute for Population Sciences (IIPS), Mumbai, to show the trend, pattern and determinants of mistimed and unwanted pregnancies. Bivariate and multinomial logistic regression model have been used with the help of Stata 13 software. The results show that the likelihood of a mistimed pregnancy is more prevalent among young women whereas the prevalence of unwanted pregnancy is observed more among the women aged 35 years or more. The results also show that the risk of experiencing mistimed pregnancy decreases if the woman belongs to ‘other’ castes and has higher education. The likelihood of unwanted pregnancy decreases among married women aged 18 years and above, those women having higher education, some autonomy and access to any mode of mass communication. Knowledge of these predictors of mistimed and unwanted pregnancy will be helpful in identifying the most vulnerable group and prioritize the intervention strategies of the reproductive health programmes for the population in need.

## Introduction

A pregnancy is said to be unintended if it is mistimed or unwanted [1]. A pregnancy is called the mistimed pregnancy when a woman did not want to become pregnant at the time of pregnancy. The pregnancies where women conceive but had no plans to become pregnant are considered as unwanted pregnancies [2]. Irrespective of their development status, women in across the globe are facing unintended pregnancies. The prevalence of unintended pregnancy can indicate the condition of women’s reproductive health and her level of autonomy in determining the number, spacing and timing of births. In 2008, out of approximately 208 million pregnancies 102 million resulted in intended births, 33 million unintended births, 41 million induced abortions, and 31 million miscarriages [3]. Even though, a decline in the unintended pregnancies has been observed, the proportion of unintended pregnancies remains high in developing countries. It is estimated that nearly two-fifths of unintended pregnancies in developing countries is either mistimed or unwanted [4].

According to the World Health Report (2005), the common cause of maternal mortality in developing countries is unwanted, mistimed and unintended pregnancy [5]. Unintended pregnancy is a threat to every sexually active woman. A common consequence of unintended pregnancy is induced abortion, which is usually unsafe in the setting where practicing abortion is illegal [6]. It is a key concern for public health as it is negatively associated with the social, economic and health issues of women and their family. The prevalence of unintended pregnancy can negatively affect maternal and child outcome such as premature birth, low birth weight, underutilization of prenatal and antenatal care, infant and child mortality, maternal mortality and unsafe abortion, maternal depression, poor psychological well-being and anxiety [7–13]. In the United States, about 50% of unintended pregnancies end due to abortion-related causes [14]. Unintended pregnancies are related to the increased risk of unfavorable prenatal and postnatal behaviors [1]. It can also have harmful consequences for the health of both mother as well as child [15].

Unintended pregnancies have many causes such as preference for sons, completed family size, lack of economic and other support from spouse [4]. In India, unwanted pregnancies are found to be significantly associated with the sex-composition of the living children [16]. Unintended pregnancies mainly result from either lack of contraceptives or inconsistent or incorrect use of contraceptive methods [17]. Available literature reveals that a wide range of factors affect unintended pregnancies. Some studies suggest that unintended pregnancies are mostly a result of non-use or incorrect use of contraceptives, or a noticeable contraceptive failure [2, 18–19]. Unintended pregnancies are associated with mother’s age and the number of previous births [2, 13, 19–21]. A study conducted in Chile reveals that women aged below 25 years and of low socioeconomic status are more likely to have an unintended pregnancy than their counterparts [22]. A study conducted in Harare also shows the significant relationship between age and unintended pregnancy. It is shown that women below the age of 19 years and more than the age of 35 years are more likely to have unintended pregnancies [23].

Including India, worldwide unintended pregnancy incidences are a major public health issue. Pregnancy intention of women at the time of pregnancy can change the maternal care behavior, and so it can eventually affect the health of their infants. Thus, there is a need to identify the risk factors of both mistimed and unwanted pregnancy as the women experiencing mistimed pregnancy may feel different towards pregnancy than those women experiencing an unwanted pregnancy.

In cultural setting like in India, post-facto rationalization does play an important role in changing the pregnancy intentions. The post—facto rationalization phenomenon is the propensity to report children as wanted when they originally were unwanted [24]. In a longitudinal study in North India, it was found that 30 percent of births were classified as unintended before childbirth, compare to 10 percent after childbirth [25]. It is documented in literatures that higher reporting of unintended pregnancies is observed among currently pregnant women than those of women having live births [26]. However, the aim of the study is to measure the trend pattern and determinants of both mistimed and unwanted pregnancies among ever-married women who were pregnant at the time of survey in India. This is one the ways to overcome or minimize the post-facto rationalization phenomenon, and hence it may not affect the responses on pregnancy intension reported by women.

## Methods and Materials

The present study has used secondary data from the National Family Health Survey (NFHS) round 1, 2 and 3 for measuring the trend and only the NFHS third round data has been utilized to determine the pattern and predictors of mistimed and unwanted pregnancy in India. The NFHS is a large scale survey coordinated by the International Institute for Population Sciences (IIPS) under the Ministry of Health and Family Welfare, Government of India. The NFHS is India’s demographic and health survey collected as part of the Demographic and Health Survey (DHS) program. The First National Family Health Survey (NFHS-1) was conducted in 1992–93, the second (NFHS-2) was conducted in 1998–99, the third (NFHS-3) was carried out in 2005–2006. This survey provides information on family planning, fertility, morbidity, nutrition, health care and HIV-related information [23]. The NFHS-3 covered a representative sample of 109,041 households out of which 124,385 were women aged between 15–49 years, and 74,369 were men aged between 15–54 years. Out of 124,385 interviewed women 5817 were ever married currently pregnant.

### Variables

#### Dependent variable

The pregnancy intention is measured on the basis of women's perception during their current pregnancy. The definitions used for measuring mistimed and unwanted pregnancy are standard and have been used in various studies [23, 27]. The mistimed and unwanted pregnancy is measured on the basis of the question: “At the time you became pregnant, did you want to become pregnant then, did you want to wait until later, or did you not want any (more) children at all?”. The options to this question were ‘Then’, ‘Later’ and ‘Not at all’. The women responded as ‘Then’ classified as wanted, ‘Later’ as mistimed and ‘Not at all’ as unwanted pregnancy.

#### Independent variable

The independent variable taken for this study were Age-group (15–24, 25–34, 35+), Caste (Scheduled Caste, Scheduled Tribe, Others caste), Religion (Hindu, Muslim, Christian and Others Religion), Place of Residence (Urban and Rural), Ideal number of children (No Child, One or two children, and 3 or more), Age at marriage (Less than 18 years and 18 years or more), Education level (No-education, Primary, Secondary and Higher), Occupation (Non-worker and Worker) and Used Method (Never used and Ever used). Woman autonomy is measured by their decision on health care seeking and on spending money. If the women have taken decision on their own or with the involvement of others, the women is said to have ‘some autonomy ‘and if the women is totally dependent on others decision, the women is said to have ‘no autonomy’ [19]. In exposure to mass media, if the women have exposure to any of the modes of mass media namely newspaper, radio, television and cinema she is said to have exposure. If she does not have exposure to these modes of mass media, then it is considered that she does not have exposure to mass media. Sex composition of living children is categorized into three categories; Sons less than daughters, Sons equal to daughters and Sons greater than daughters.

### Statistical Analysis

Univariate and bivariate analysis have been used to show trend and pattern of mistimed and unwanted pregnancy of women having different background characteristics. Chi square test of significance is used to show the association between dependent and independent variables. For multivariate analysis, a multinomial logistic regression has been used. In the multinomial logistic regression analysis wanted pregnancy is taken as baseline outcome. The whole analysis has been carried out in STATA-13 software.

## Results

**Fig 1:**
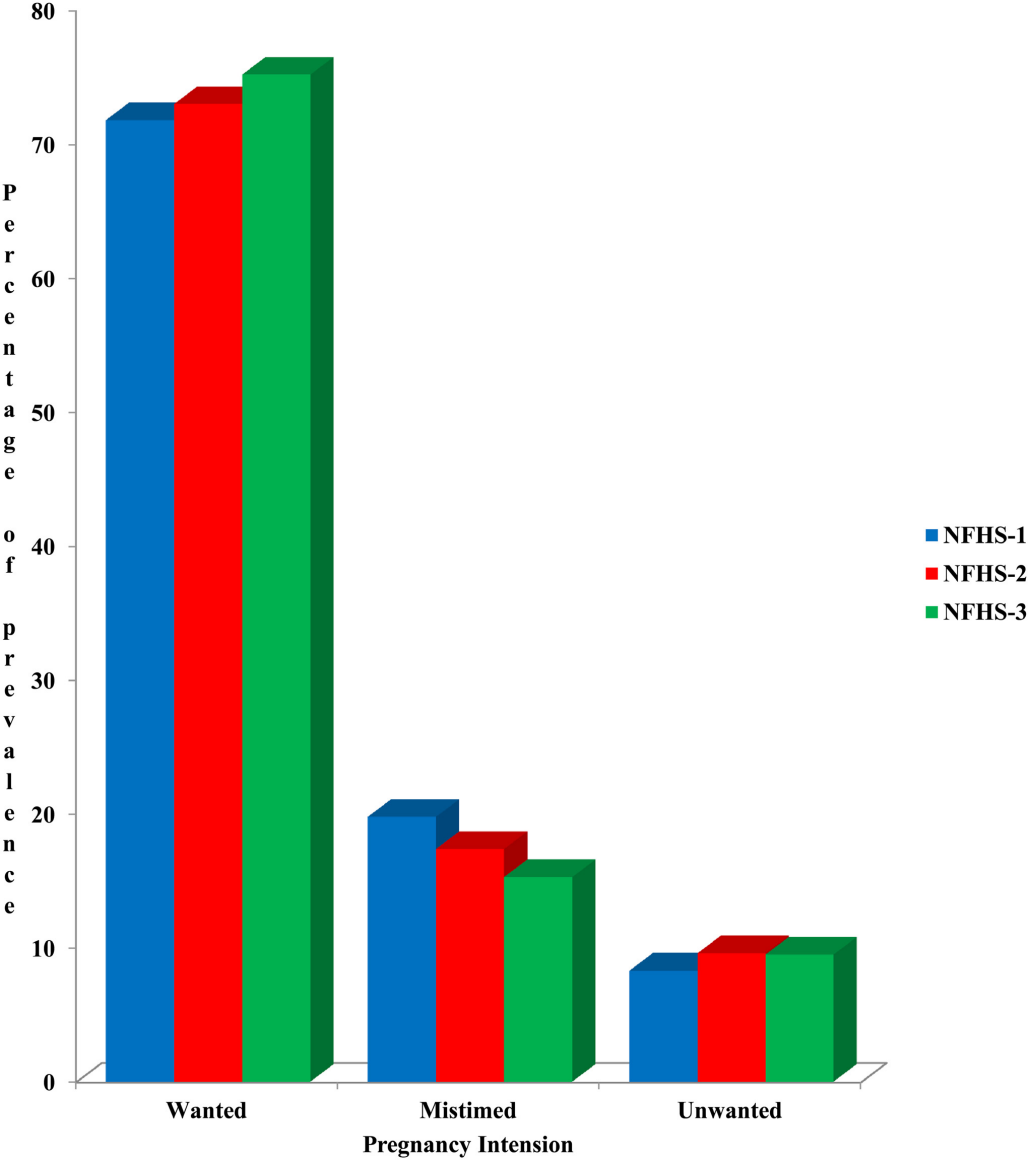
Trend of pregnancy intention among currently pregnant married women.

The Fig 1 is showing the trend of wanted, mistimed and unwanted pregnancy in India during NFHS-1 NFHS-2 and NFHS-3. The Fig 1 is showing an increase in the percentage of wanted pregnancy from NFHS-1 to NFHS-3. The level of mistimed pregnancy has decreased from nearly 20% to 15.3% though the level of unwanted pregnancy has increased during first to third round of the survey.

### Pattern and Differentials of Unintended Pregnancy

The Table 1 gives the percent distribution of the mistimed and unwanted pregnancy by background characteristics of women. It shows the significant association between pregnancy intension and background characteristics such as age-group, religion, ideal number of children, age at marriage, education level, exposure to mass media, used method and sex-composition of surviving children. It depicts that wanted and mistimed pregnancy decreases by the age of women whereas unwanted pregnancy increases. The wanted pregnancy is the lowest (74.7%) among scheduled caste women. The mistimed pregnancy is highest (15.5%) among “Others” caste whereas unwanted pregnancy is highest (10.7%) among scheduled caste women. The Muslim religion accounts for lowest (68.7%) level of wanted pregnancy and the highest proportion of both mistimed (18.2%) and unwanted (13.1%) pregnancies. According to the place of residence, there is not much difference in the level of mistimed and unwanted pregnancy. The women who reported their ideal number of children is one or two children, the mistimed pregnancy, is more among them whereas unwanted pregnancy is more prevalent among those who reported three or more ideal number of children.

**Table 1:** Number and Percent distribution of pregnancy intentions by women’s characteristics Pregnancy Intention n (%).

	Wanted	Mistimed	Unwanted	P value
**Age-group**				0
15–24	2635 (77.9)	592 (17.5)	156 (4.6)	
25–34	1542 (71.2)	266 (12.3)	357 (16.5)	
35+	175 (65.2)	9 (3.5)	84 (31.3)	
**Caste**				0.145
Scheduled Caste	775 (74.7)	150 (14.5)	112 (10.8)	
Scheduled tribe	670 (77.4)	130 (15.0)	66 (7.6)	
Others	2939 (75.1)	607 (15.5)	368 (9.4)	
**Religion**				0
Hindu	3105 (76.6)	596 (14.7)	353 (8.7)	
Muslim	700 (68.7)	185 (18.2)	133 (13.1)	
Christian	372 (74.6)	89 (17.8)	38 (7.6)	
Others	193 (78.8)	28 (11.6)	24 (9.6)	
**Place of residence**				0.636
Urban	1682 (75.0)	343 (15.3)	217 (9.7)	
Rural	2692 (75.3)	547 (15.3)	336 (9.4)	
**Ideal number of Children**				0
No child	28 (93.8)	1 (3.1)	1 (3.1)	
One or two child	2847 (76.7)	612 (16.5)	252 (6.8)	
3 or more	1506 (72.6)	278 (13.4)	291 (14.0)	
**Age at marriage**				0
Less than 18	1937 (74.2)	376 (14.4)	298 (11.4)	
18 or more	2456 (76.6)	526 (16.4)	224 (7.0)	
**Education level**				0
No education	1622 (75.6)	272 (12.7)	251 (11.7)	
Primary	586 (71.0)	155 (18.8)	84 (10.2)	
Secondary	1749 (74.6)	429 (18.3)	166 (7.1)	
Higher	434 (86.5)	47 (9.4)	21 (4.1)	
**Women Autonomy**				0.117
Some	2738 (74.7)	531 (14.5)	396 (10.8)	
No	1573 (73.1)	329 (15.3)	250 (11.6)	
**Occupation**				0.739
Non worker	3033 (75.7)	629 (15.7)	345 (8.6)	
Worker	1375 (75.9)	270 (14.9)	167 (9.2)	
**Exposure to mass-media**				0
No	978 (74.1)	169 (12.8)	173 (13.1)	
Yes	3409 (75.8)	738 (16.4)	351 (7.8)	
**Used Method**				0
Never	3216 (78.6)	579 (14.1)	299 (7.3)	
Ever	1192 (69.2)	319 (18.5)	212 (12.3)	
**Sex Composition**				
Sons less than daughters	1260 (72.9)	287 (15.3)	189 (11.8)	0
Sons equal to daughters	2266 (82.6)	331 (12.1)	135 (5.3)	
Sons greater than daughters	882 (64.1)	280 (21.4)	187 (14.5)	

The proportion of mistimed pregnancy is less among women married before 18 years whereas unwanted pregnancy is more (11.4%) among women married before 18 years. The education level of women and the nature of pregnancies are strongly linked. The results show that as the education level of the women increases the prevalence of both mistimed and unwanted pregnancies decreases.

The women having some autonomy are experiencing fewer burdens of both mistimed (14.5%) and unwanted (10.8%) pregnancy than the women having no autonomy. The less (14.9%) proportion of mistimed pregnancy is observed among working women though the unwanted pregnancy is higher (9.2%) among them. The women having exposure to any mode of mass media, the proportion of mistimed pregnancy is slightly higher (16.8%) and the unwanted pregnancy is far lower (7.8%) than those who did not expose to any mass media. The prevalence of both mistimed and unwanted pregnancy is more among women ever used any method of contraception. The prevalence of both mistimed (21.4%) and unwanted (14.5%) pregnancies is higher among women who have number of sons greater than number of daughters.

### Factors Affecting Unintended Pregnancy

The correlates of mistimed and unwanted pregnancy are shown in the Table 2. Age-group of women is significantly associated with the both mistimed and unwanted pregnancy. The women in the age-group 25–34 years are at 35% lower risk of having mistimed pregnancy whereas 3.7 times more likely to have unwanted pregnancy than the women in the age-group 15–24. The women aged 35 or more are 53% less likely to have mistimed pregnancy but 7.7 times more likely to have unwanted pregnancy than the women in the age-group 15–24 years. The women of “other” castes are 20% less likely to have mistimed pregnancy than the scheduled castes though the relationship is not highly significant. The women of the Christian religion are nearly 1.4 times significantly more likely to have mistimed pregnancy than the women of Hindu religion.

**Table 2:** Multinomial logistic regression of factors associated with mistimed and unwanted pregnancies among currently pregnant ever married women in India.

	Mistimed		Unwanted	
Variable	RRR	95% CI	RRR	95% CI
**Age-group** ^®^				
15–24	1		1	
25–34	0.646***	(0.52–0.8)	3.695***	(2.78–4.92)
35+	0.469***	(0.28–0.79)	7.733***	(4.99–11.98)
**Caste** ^®^				
Scheduled Caste	1		1	
Scheduled Tribe	1.109	(0.78–1.57)	0.887	(0.56–1.4)
Others	0.799*	(0.62–1.03)	1.014	(0.74–1.39)
**Religion** ^®^				
Hindu	1		1	
Muslim	1.152	(0.87–1.53)	1.062	(0.76–1.47)
Christian	1.39*	(0.97–1.98)	0.698	(0.42–1.17)
Others	0.795	(0.49–1.28)	1.059	(0.6–1.87)
**Place of residence** ^®^				
Urban	1		1	
Rural	1.017	(0.82–1.26)	0.893	(0.68–1.17)
**Ideal number of Children** ^®^				
No child	1		1	
One to two children	1.334	(0.38–4.69)	1.158	(0.25–5.33)
3 or more	1.318	(0.38–4.61)	1.159	(0.25–5.3)
**Age at marriage** ^®^				
Less than 18	1		1	
18 or more	1.104	(0.89–1.37)	0.538***	(0.41–0.71)
**Education level** ^®^				
No education	1		1	
Primary	1.326*	(0.98–1.79)	1.429**	(1.01–2.02)
Secondary	1.343**	(1.04–1.74)	0.863	(0.62–1.2)
Higher	0.64**	(0.4–1.02)	0.27***	(0.14–0.53)
**Women Autonomy** ^®^				
Some	1		1	
No	1.438	(0.89–2.34)	2.051**	(1.16–3.64)
**Occupation** ^®^				
Non worker	1		1	
Worker	1.003	(0.81–1.25)	0.795*	(0.61–1.04)
**Communication** ^®^				
No	1		1	
Yes	0.945	(0.74–1.21)	0.623***	(0.47–0.83)
**Used any method** ^®^				
Never	1		1	
Ever	1.435***	(1.17–1.76)	1.572***	(1.22–2.02)
**Sex Composition** ^®^				
Sons less than daughters	1		1	
Sons equal to daughters	0.558***	(0.44–0.71)	0.779	(0.58–1.05)
Sons greater than daughters	1.128	(0.89–1.43)	1.516***	(1.15–2)

Note:

^®^ Reference category,

* (p<0.10),

** (p<0.05),

*** (p<0.01),

RRR = Relative risk ratio; CI = Confidence interval

Age at marriage is significantly associated with unwanted pregnancy yet not associated with mistimed pregnancy. The women married after 18 years are 46% less likely to have unwanted pregnancy than the women married before 18 years.

The higher education of women has shown the significant impact on the mistimed and unwanted pregnancies. It remarkably reduces the chances of having mistimed and unwanted pregnancy. The women having higher education are 36% less likely to have mistimed pregnancy and 73% less likely to have an unwanted pregnancy. The women autonomy is significantly associated with unwanted pregnancy. The women having no autonomy are approximately 2.1 times more likely to have unwanted pregnancy than the women having some autonomy. The working women are 20% less likely to have unwanted pregnancy than the women not working for cash or kind in return.

The women having exposure to mass media are 38% less likely to have unwanted pregnancy than the women having no exposure to any source of mass media. The women ever used any method of contraception are 1.4 times and 1.6 times more likely to have mistimed and unwanted pregnancy respectively. Sex-composition of surviving children has shown to have significant impact on both mistimed and unwanted pregnancy. The women having sons and daughters in equal number are 41% less likely to have mistimed pregnancy than the number of sons less than daughters. The likelihood of experiencing unwanted pregnancy increases by nearly 1.5 times if the women have more number of sons than daughters with respect to those women who had less number of sons than daughters.

## Discussion

The mistimed and unwanted pregnancy is a major public health concern as it is linked to the negative health outcomes of both women and children. The measures and program strategies should be pursued to minimize the level of mistimed and unwanted pregnancies. The prevalence of mistimed and unwanted pregnancy can be reduced by understanding the risk factors through proper interventions. In the present study, the trend, pattern and determinants of mistimed and unwanted pregnancy has been measured among currently pregnant ever-married women in India.

Results show that nearly one-fourth of the pregnancies was unintended (mistimed and unwanted) in India. The trend shows a slight decline in the mistimed pregnancies whereas the level of unwanted pregnancy has increased from NFHS-1 to NFHS-3.

It is observed that various factors affecting mistimed and unwanted pregnancy among currently pregnant women. The women in the younger age-group are more likely to have mistimed pregnancy whereas the likelihood of unwanted pregnancy is more among older women. This finding is also supported by the previous studies conducted in Ethiopia, Tanzania and Kenya [9, 28–29]. Low age at marriage is found to be a contributing factor for unwanted pregnancy. Evidence also suggests that young married women are at the higher risk of experiencing early and unplanned pregnancy [30].

Education status of women acts as a major predictor of unintended pregnancy [18, 31–32]. In the present study, the likelihood of both mistimed and unwanted pregnancy is lower among higher educated women. It may be because women having higher education are more aware of an effective family planning methods and their correct use [16].

The women with some autonomy and having exposure to any mode of mass media are also less likely to experience an unwanted pregnancy. The similar results can be corroborated by previous findings in the district in Southern Ethiopia [32]. The women having some autonomy can make a decision on spacing, number and timing of their children. The focus on increasing women autonomy can decrease both the mistimed and unwanted pregnancy significantly.

Similar to previous studies in Ethiopia, Malawi and Nepal [20, 29, 33], the present study have found significant association between women using contraceptives under 'ever used' category and wanted pregnancy. The study reveals that women using contraceptives under the 'ever used' category are more likely to have both mistimed and unwanted pregnancies. The possible reasons could be barriers to accessing the contraceptive methods, lack of knowledge about correct use, misconception about methods and discontinuation are possible factors affects the use of contraception [20, 33]. In addition, women in ‘ever used’ of contraception categories may have higher fecundity than those who were in ‘never used’ category. The study conducted in India revealed that nearly two fifths of ever-married women discontinued contraception because of the method failure [34]. This study also finds a significant association between the sex composition of children and mistimed and unwanted pregnancy. In case of unwanted pregnancy, the association is showing clear evidences of son preference in women’s fertility intension too. The women having more number of sons than daughters are more likely to have unwanted pregnancy than the women who have more number of daughters than sons. The result indicates that if the desired number of son is attained the desire for the other births reduces dramatically, and there is also a possibility of excess fertility [35].

## Conclusion

Nearly one-fourth of the currently pregnant ever-married women experienced unintended pregnancy in India. This study has found various factors affecting mistimed and unwanted pregnancy yet the factors are not similar for both. The likelihood of mistimed pregnancy is more among young women whereas the unwanted pregnancy is more among aged women of 35 years or more. The risk of experiencing mistimed pregnancy decreases if the woman belongs to ‘others’ caste and has attained higher levels of education. The likelihood of unwanted pregnancy decreases if the women married after 18 years of age, higher educated, having some autonomy and have access to any mode of mass communication. Knowledge of these predictors of mistimed and unwanted pregnancy will be helpful in identifying the vulnerable groups and prioritizing the intervention strategies through appropriate modifications in the reproductive health programs and policies.
